# Mouse Models of Nonalcoholic Steatohepatitis: Head-to-Head Comparison of Dietary Models and Impact on Inflammation and Animal Welfare

**DOI:** 10.1155/2020/7347068

**Published:** 2020-07-13

**Authors:** Andreas Kroh, Vanina Ivanova, Hannah Drescher, Julia Andruszkow, Thomas Longerich, Jochen Nolting, Roman Eickhoff, D. Heise, Karl P. Rheinwalt, Ulf P. Neumann, Florian T. Ulmer

**Affiliations:** ^1^Department of General, Visceral and Transplantation Surgery, RWTH Aachen University Hospital, 52074 Aachen, Germany; ^2^Department of Internal Medicine III, RWTH Aachen University Hospital, 52074 Aachen, Germany; ^3^Institute of Pathology, RWTH Aachen University Hospital, 52074 Aachen, Germany; ^4^Institute of Pathology, University Hospital Heidelberg, 69120 Heidelberg, Germany; ^5^Department for Bariatric and Metabolic Surgery, St. Franziskus-Hospital, 50825 Cologne, Germany; ^6^Department of Surgery, Maastricht University Medical Center, 6202 AZ Maastricht, Netherlands

## Abstract

A variety of dietary nonalcoholic steatohepatitis (NASH) mouse models are available, and choosing the appropriate mouse model is one of the most important steps in the design of NASH studies. In addition to the histopathological and metabolic findings of NASH, a sufficient mouse model should guarantee a robust clinical status and good animal welfare. Three different NASH diets, a high-fat diet (HFD60), a western diet (WD), and a cafeteria diet (CAFD), were fed for 12 or 16 weeks. Metabolic assessment was conducted at baseline and before scheduled sacrifice, and liver inflammation was analyzed via fluorescence-associated cell sorting and histopathological examination. Clinical health conditions were scored weekly to assess the impact on animal welfare. The HFD60 and WD were identified as suitable NASH mouse models without a significant strain on animal welfare. Furthermore, the progression of inflammation and liver fibrosis was associated with a decreased proportion of CD3^+^ NK1.1^+^ cells. The WD represents a model of advanced-stage NASH, and the HFD60 is a strong model of nonalcoholic fatty liver disease (NAFLD) and metabolic syndrome. However, the CAFD should not be considered a NASH model.

## 1. Introduction

Due to the increasing prevalence of obesity, the incidence of nonalcoholic fatty liver disease (NAFLD), which is the hepatic manifestation of metabolic syndrome, has also increased [1, 2]. NAFLD encompasses a variety of pathologies that range from simple hepatic steatosis to nonalcoholic steatohepatitis (NASH) [3], which can progress and result in cirrhosis, hepatocellular carcinoma, and end-stage liver disease [4].

In recent years, several new rodent models resembling the pathogenesis of human NAFLD/NASH have been described. The different rodent models can be classified into two large groups. The first includes models in which the disease is acquired after dietary or pharmacological manipulation, and the second includes genetically modified models in which NASH develops spontaneously. Models with genetic alterations allow information on the role of single proteins, hormones, and receptors in NASH pathology but may not reflect human disease appropriately. Diet-induced models are usually based on ad libitum feeding of diets enriched with various combinations of fat, cholesterol, and sugars or involve feeding nutrient-deficient diets such as the methionine and choline-deficient diet (MCD). Unfortunately, the MCD does not induce features of the metabolic syndrome and affects animal welfare [5]. On the other hand, overnutrition-based models have demonstrated substantial metabolic similarity to humans with NASH, but with variable reproducibility of the histological features of NASH [6].

Emerging evidence suggests that NASH pathogenesis depends on the complex interaction and cross-talk between environmental influences and host immune system and involves “multiple hits” [4, 7]. To date, no single rodent model has encompassed the full spectrum of human disease progression, but individual models can imitate particular characteristics of human disease progression. Incorrect model selection results in invalid data and the waste of laboratory animals, which must be avoided in accordance with the “3 Rs” (Replacement, Reduction, and Refinement) tenet.

Poor documentation and missing reports about the impact on clinical status and animal welfare in the current literature hamper the quality of NASH research. A recent review of mouse models of NASH demonstrates an inconceivable lack of information about the liver injury and basic metabolic and clinical condition in NASH research and suggests minimal criteria for rodent NASH models [8]. All NASH models should at least report weight gain, metabolic condition, liver injury, and liver inflammation assessed by an experienced liver pathologist. Furthermore, documentation of animal welfare status and adherence to the ARRIVE guidelines is crucial and will improve the quality of recent and future NASH research.

In our study, we compare a high-fat diet, a western diet, and a cafeteria diet in detail to identify valid NASH mouse models for further intervention studies for NASH such as bariatric surgery or drug interventions. Due to the above-mentioned limitations of genetically modified mice, we focused on C57/Bl6 mice as the most widely used genetic background as a model for human diseases. Furthermore, for the first time, clinical conditions and an animal welfare assessment were evaluated to provide reliable data collection and better documentation of animal health status.

## 2. Materials and Methods

### 2.1. Animals

All animal experiments were approved by the governmental care and use committee (LANUV), Recklinghausen, NRW, Germany, granted official permission (84-02.04.2014.A356), and conducted in accordance with the federal German law and European directive 2010/63/EU on the protection of animals used for scientific procedures. Our experiments were also in compliance with the Guide for the Care and Use of Laboratory Animals (8th edition, NIH publication, 2011, USA). Male C57/Bl6 J mice were purchased from Charles River (Charles River Laboratories, Inc., Germany) at the age of four weeks. Female mice were excluded due to sex differences in obesity-induced complications. Animals were housed under SPF conditions according to FELASA guidelines (http://www.felasa.org) on a 12-hour light/dark cycle and were acclimated to the RWTH Aachen animal house facility on standard chow (SC, V1534-300, ssniff GmbH, Germany) for two weeks before assignment to one of the four experimental diet groups.

### 2.2. Study Design

Mice were placed on a standard chow diet as a control (V1534-300, ssniff GmbH, Germany). To induce NASH and metabolic syndrome, mice received sucrose-enriched water (10%) and either a high-fat diet with 60 kcal% fat (HFD60, D12492, Research Diets, USA), a western diet with 40 kcal% fat, 20 kcal% fructose, and 2 kcal% cholesterol (WD, D09100301, Research Diets, Inc., New Brunswick, NJ, USA), or a cafeteria diet (CAFD) at six weeks of age. The CAFD mice were fed SC ad libitum in addition to five high-caloric human snack foods that changed daily. These human snacks included cookies, processed meat, nuts, candy bars, and peanut chips and were provided in excess following a fixed timetable (Supplementary Table 1). Diets were fed in two independent studies for 12 weeks (SC, HFD60, WD, and CAFD) and for 16 weeks (SC, HFD60, and WD) with six animals in each group. Snack intake was measured daily, and weight and diet intake were measured weekly. After 12 and 16 weeks, the mice were euthanized, and the livers and gonadal fad pads were removed for further investigation. Furthermore, blood analysis and intraperitoneal glucose tolerance test (IP-GTT) were conducted twice, once before starting the diet (baseline) and once before scheduled sacrifice.

### 2.3. Clinical Status

All animals were monitored daily, and clinical status was scored weekly by an experienced technician. A clinical scoring system adapted from Kanzler et al. was used for animal welfare assessment [9]. Categories encompassed general state, behavior, clinical results, and trial-specific indicators. General state involved the assessment of body weight, fur defects, stoma, and eyes. Nutrition, social behavior, and activity were categorized under behavior and digestion, pulse, respiration, and vegetative symptoms under clinical results. Due to the crucial role of the liver in this experiment, trial-specific indicators included parameters associated with liver failure. Different parameters within categories were documented and scored depending on clinical impact and assessed in a range from 1 to 20 points. Points of all parameters were summed, and mice were classified into five “degree of strain (DS)” groups. DS0 indicated no alteration and a good physiological clinical status, whereas DS1-DS4 represented minor, moderate, critical, or high-grade strain categories. Depending on the degree of strain, the score sheet indicates specific interventions, such as closer monitoring, consultation of a veterinarian, supportive measures, or termination of the experiment. The design of the score sheet including the DS is depicted in Tables 1 and 2.

### 2.4. IP-GTT and HOMA-IR

Mice were fasted overnight and given an intraperitoneal glucose injection (2 g/kg, D-glucose) after their fasting blood glucose levels were measured. Blood glucose was measured in whole venous blood from the tail vein (Accu-Chek Aviva, Roche Diabetes Care Deutschland GmbH, Germany) at 0 (fasting), 30, 90, 120, 150, and 180 minutes after glucose administration. To compare glucose tolerance, the area under the glucose concentration-time curve was analyzed. Insulin resistance was calculated using the Homeostasis Model of Insulin Resistance (HOMA-IR) index: (fasting insulin (mU/l) × fasting glucose (mmol/l))/22.5 [10].

### 2.5. Biochemical Analysis

Blood samples were collected and centrifuged in heparin-embedded tubes. As an index of liver damage and for further metabolic assessment, serum transaminase (ALT), cholesterol, and triglyceride levels were measured according to the standard procedures of the Laboratory Facility of the Institute of Laboratory Animal Science at the University Hospital RWTH of Aachen.

### 2.6. Histopathological Examination

Liver biopsies were collected, and the median lobe was fixed in 4% paraformaldehyde. Then, liver tissue was embedded in paraffin, and four-micrometer-thick whole tissue sections were cut. To assess hepatic morphology and fibrosis, sections were stained with hematoxylin and eosin (H&E), periodic acid-Schiff (PAS), and reticulin. Histological assessment and scoring were performed by two pathologists blinded to the study conditions. The Nonalcoholic Fatty Liver Disease Scoring System (NAS) was used to quantify steatosis (0-3), lobular inflammation (0-3), and hepatocellular ballooning (0-2) as described elsewhere [11]. Liver biopsies scored NAS ≥ 5 were classified as definitive NASH [12]. Fibrosis of liver samples was classified using the clinical criteria outlined by Kleiner et al. [13]. In addition to the calculation of the fibrosis score (reticulin staining), Sirius red staining was performed to assess collagen fibers as previously described [14]. F4/80^+^ macrophages were detected by an F4/80 monoclonal antibody (BM8, 14-4801-82, eBioscience™, Austria) and neutrophils by a purified anti-mouse Ly-6G antibody (1A8, 127601, BioLegend, USA). Photomicrographs of F4/80, Ly6G, and Sirius red staining were taken at 200x magnification and analyzed using the open source software ImageJ.

### 2.7. Fluorescence-Activated Cell Sorting (FACS)

For FACS analysis, the left liver lobes were minced, digested for 30 minutes with collagenase IV (Worthington) at 37°C, homogenized, and then filtered through 70 *μ*m cell strainers. Afterwards, density gradient centrifugation (LSM-1077, PAA) was performed at 812 x g/23°C for 20 minutes on the resulting cell suspension. Subsequently, leukocytes were taken from the interphase layer, washed twice with Hank's balanced salt solution containing 0.5% bovine serum albumin and 2 mM ethylenediaminetetraacetic acid, and then subjected to flow cytometric analysis.

Isolated leukocytes were stained with combinations of the following monoclonal antibodies: CD45 and Ly6G (both BD, Germany); CD8a and F4/80 (both BioLegend, USA); CD4, CD11b, and NK1.1 (all eBioscience™, Austria); and CD3 (Miltenyi, Germany). Flow cytometry was performed on an LSR Fortessa (BD Biosciences), and data were analyzed with FlowJo (TreeStar, USA).

### 2.8. Statistics

Sample size was calculated using SAS 9.3 (SAS, Cary, USA) adopting a significance level of 0.05 and a power of 80%. With an expected drop-out rate of 10%, the final sample size was *n* = 6. The distribution of variables was analyzed using the Shapiro-Wilk normality test. Continuous data are presented as the mean and standard deviation (SD). In cases of normal distribution, significant differences between groups were determined by ordinary one-way or two-way ANOVA and Tukey's post hoc multiple comparison test. In the case of nonnormal data distribution, the Kruskal-Wallis test was used with Dunn's multiple comparison test. *p* < 0.05 was considered to indicate statistical significance. Statistical analysis and graphical representations were carried out using GraphPad Prism (version 7.0, La Jolla, USA).

## 3. Results

The control diet, HFD60, and WD were provided for 12 and 16 weeks. Metabolic assessment after 12 weeks of CAFD showed significantly less weight in comparison to those of HFD60 and WD and no significant liver inflammation in comparison to control animals (Supplementary Figure 1). Consequently, CAFD is inferior to HFD60 and WD as a valid NASH mouse model. Following the reduction principle within the 3Rs of animal research, CAFD was not provided for 16 weeks.

### 3.1. Body Weight and Food Intake

**Figure 1:**
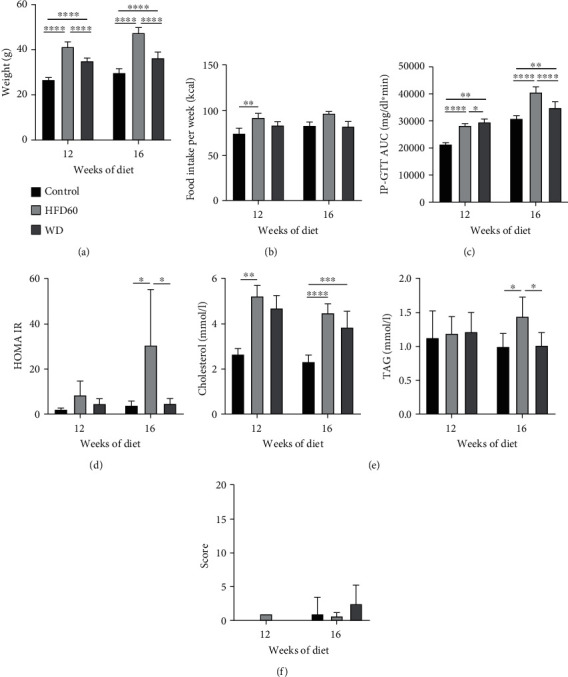
Metabolic assessment clinical status. (a) Body weight at 12 weeks and 16 weeks showed a significant gain in total body weight of WD and HFD60 mice compared to that of control animals. (b) Food intake per week for 12 weeks and 16 weeks showed a significantly higher energy intake in HFD60 mice than that in other groups after 12 weeks of diet. (c) Area under the glucose time curve (AUC) after dietary treatment for 12 or 16 weeks. HFD60 and WD animals displayed significantly impaired glucose tolerance, which is depicted as an increase in IP-GTT AUC compared to chow-treated animals. Comparing HFD60 and WD mice, impaired glucose tolerance was more pronounced after 16 weeks in HFD60 mice. (d) HOMA-IR at 12 weeks and 16 weeks displayed insulin resistance in HFD60 mice compared to control mice but no difference between control and WD mice after 12 and 16 weeks. (e) Triglyceride (TAG) and cholesterol levels at 12 weeks and 16 weeks showed increased cholesterol levels in HFD60 and WD mice after 12 and 16 weeks. TAG levels were only increased significantly in HFD60 mice compared to control and WD mice after 16 weeks of diet. (f) Clinical scores for 12-week and 16-week studies display no or only mild impairment with a maximum DS1. Mild impairment was based on fur defects due to fighting animals. After separating the animals, the fur defects ceased. ^∗^*p* < 0.05, ^∗∗^*p* < 0.01, ^∗∗∗^*p* < 0.001, and ^∗∗∗∗^*p* < 0.0001.

Final body weight was significantly higher in the HFD60 and WD groups than in the control group after 12 and 16 weeks of diet (Figure 1(a)). Comparing both NASH diets, final body weight was significantly higher in the HFD60 group than in the WD group (Figure 1(a)). Interestingly, we observed a tendency toward pronounced caloric intake in HFD60 mice with significant differences in 12-week-old animals, but WD mice did not show any difference from control mice (Figure 1(b)).

### 3.2. Metabolic Assessment

As expected, 12 weeks of HFD60 and WD significantly impaired glucose tolerance as measured by the IP-GTT (Figure 1(c)). After 16 weeks of diet, glucose tolerance was still impaired in both NASH groups, but glucose intolerance was even more pronounced in HFD60 mice than in WD mice (Figure 1(c)). Additionally, insulin resistance was apparent in HFD60 mice after 12 and 16 weeks but not in WD mice (Figure 1(d)).

As another component of metabolic syndrome, cholesterol serum levels were increased in HFD60 and WD mice compared to those in the control group, with the HFD60 group achieving statistical significance after 12 and 16 weeks and the WD group after 16 weeks only (Figure 1(e)). Serum triglyceride levels did not differ between groups after 12 weeks of diet but were significantly increased in the HFD60 group compared with the other groups after 16 weeks (Figure 1(e)).

### 3.3. Clinical Status

We could not identify any differences between groups regarding the clinical score or the DS (Figure 1(f)). There were only minor deviations from normal status and behavior. Defects of fur were observed in two cages due to fighting mice. These mice were classified as DS 1 due to the fur defect and aggressive social behavior. However, after the mice were separated, the fights stopped, and the fur defects ceased. In the 16-week study, 66% of the HFD60 group and 50% of the WD group showed greasy, shiny fur from the 15th week onward. The coat remained smooth, and changes did not progress over the course of the experiment. Apart from this exception, the general and clinical state was not affected, and all mice showed normal social behavior and no signs of any strain.

### 3.4. Liver Inflammation and Fibrosis

**Figure 2:**
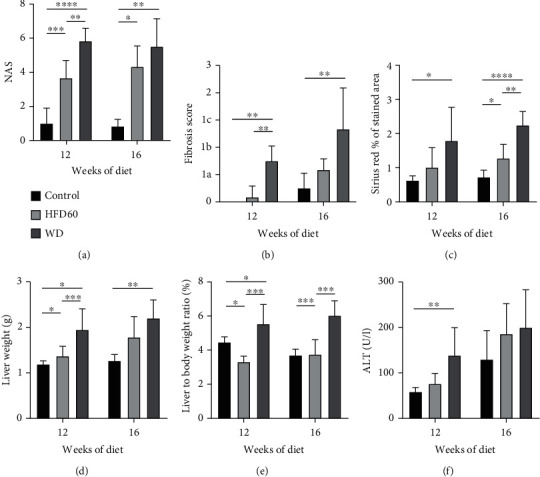
Liver inflammation and fibrosis. (a) NAS scores for 12 weeks and 16 weeks; NAS was significantly higher in the WD and HFD60 groups than in the control group after 12 and 16 weeks and was higher in WD mice than in HFD60 mice after 12 weeks. Notably, NAS ≥ 5 in each individual animal after 12 weeks of WD. (b) The fibrosis score was increased in WD animals compared to that of control and HFD60 at both timepoints. (c) Sirius red staining displayed a significantly higher proportion of connective tissue fibers in WD livers than in control livers at the 12-week and than in control and HFD60 livers at the 16-week timepoint. At the 16-week timepoint, HFD60 showed significantly more connective tissue fibers compared to the control group. (d) Liver weight and (e) liver-to-body weight ratio were significantly increased in WD mice compared with those of mice in the other groups after 12 and 16 weeks of diet. (f) Significantly increased ALT levels in WD mice showed enhanced liver injury in WD mice after 12 weeks of diet.

**Figure 3:**
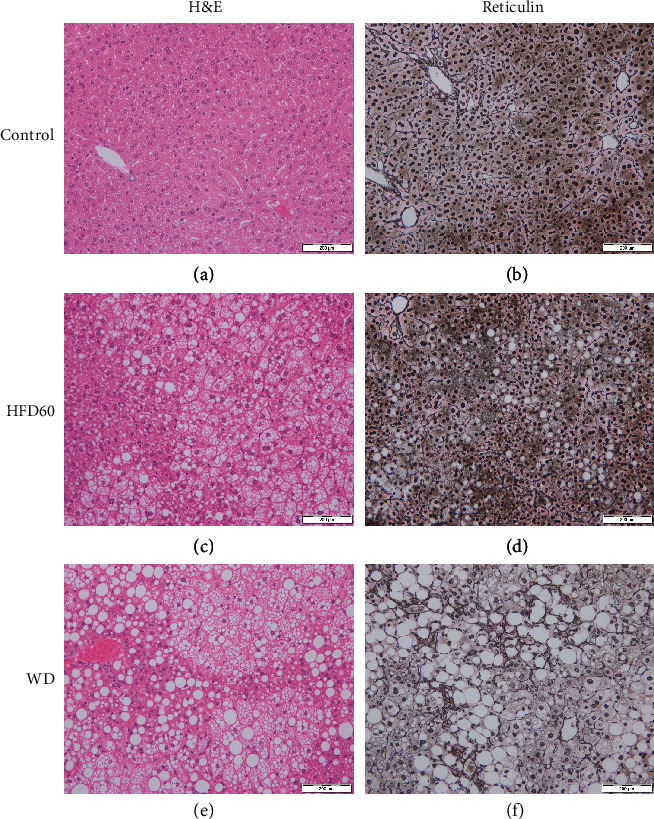
H&E- and reticulin-stained liver sections. Representative histological images of (a, b) control, (c, d) HFD60, and (e, f) WD mice at 12 weeks. Images show severe steatosis, especially in HFD60 livers, and fibrosis after WD treatment. Magnification 200x, scale bars 200 *μ*m.

After 12 weeks of diet, the NAS was significantly higher in the WD livers than in control and HFD60 livers. The mean value of the WD group was 5.833 ± 0.7528, and the NAS of each individual mouse was ≥5, resulting in the diagnosis of definitive NASH in each WD mouse. Moreover, NAS in HFD60 animals was significantly higher than that in control animals. At 16 weeks, the WD group's NAS was still significantly higher than that in the control group, but not every animal consistently reached a NAS ≥ 5 (Figure 2(a)). Regarding the liver fibrosis score, the WD-treated groups showed more fibrosis than the HFD60 and control groups with scores that were significantly different from those of both the HFD60 and control groups after 12 weeks and from only those of the control group after 16 weeks of diet (Figure 2(b)). Representative histological images of H&E- and reticulin-stained liver sections depict severe steatosis in the HFD60 and WD groups as well as fibrosis after WD treatment (Figure 3).

**Figure 4:**
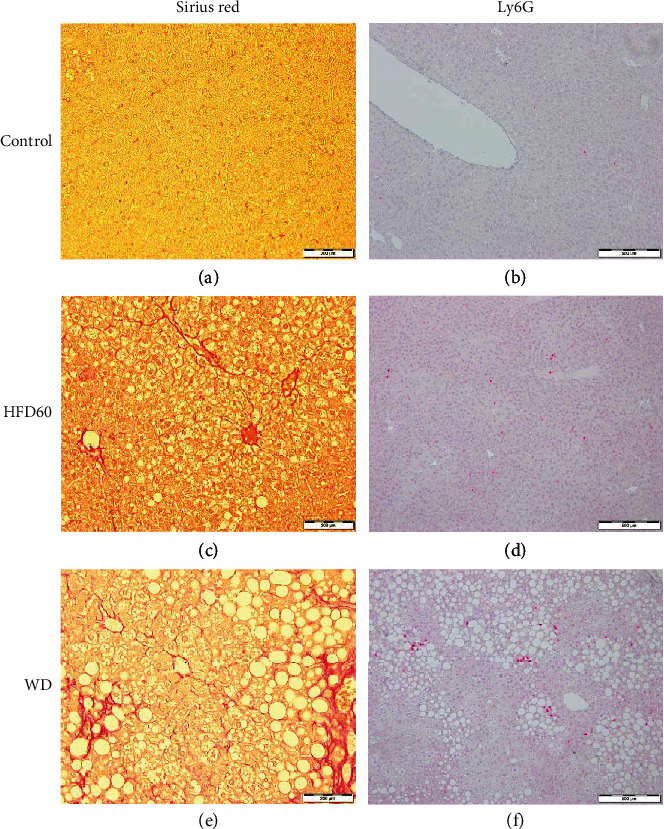
Sirius red and Ly6G staining. Representative histological images of (a, b) control, (c, d) HFD60, and (e, f) WD mice at 16 weeks for Sirius red and 12 weeks for Ly6G. (a, c, and e) Sirius red staining, magnification 200x, scale bars 200 *μ*m, (b, d, and f) Ly6G, magnification 100x, scale bars 100 *μ*m; Sirius red images show fibrosis, especially pronounced in WD (a) and less in HFD60 (c) livers; Ly6G staining reveals clusters of neutrophils in WD-fed mice (b) and more scattered in HFD60 mice (d). Control livers only presented with a few isolated neutrophils.

To support the results of the fibrosis score, we performed Sirius red staining (Figures 4(a), 4(c), and 4(e)). The proportion of connective tissue fibers was significantly greater in WD livers than in control livers after 12 weeks; after 16 weeks, the WD livers showed a greater proportion of fibers than the control and HFD60 livers. Additionally, HFD60 livers showed more collagen fibers than control livers at the 16-week timepoint (Figure 2(c)).

Both liver weight and liver-to-body weight ratio were significantly higher in WD animals than in control and HFD60 animals after 12 weeks of diet. At 16 weeks, only the difference between WD and control mice remained significant (Figures 2(d) and 2(e)). To further characterize hepatocellular injury, serum alanine aminotransferase (ALT) levels were analyzed. In contrast to the 16-week study, 12-week results showed a significant difference between the WD and the control group (Figure 2(f)). Taking these results into account, the severity of hepatocellular injury correlates with the degree of liver inflammation (NAS). However, due to a considerable standard deviation of the 16-week data set, these results should be interpreted cautiously (Supplementary Figure 2).

**Figure 5:**
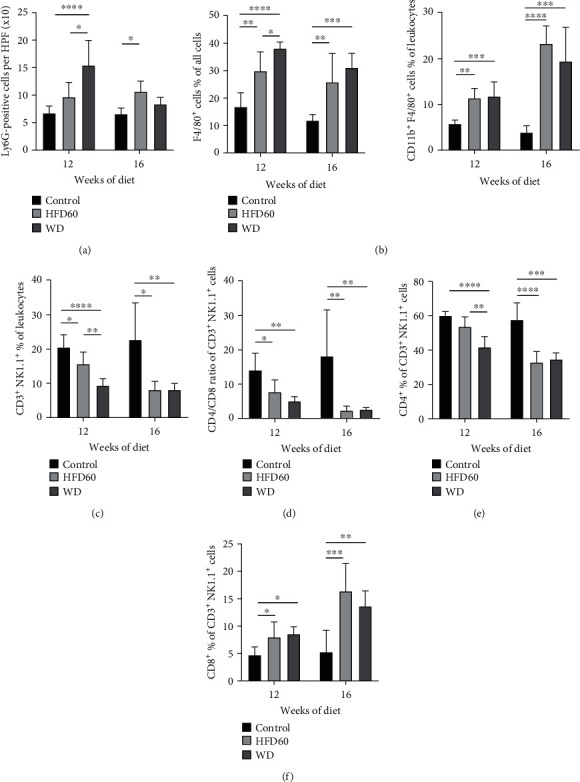
Inflammatory response. (a) Ly6G^+^ cells per HPF display a significant difference between WD and control as well as HFD60 and control at 12 weeks and only between HFD60 and control at 16 weeks. (b) F4/80^+^ cells in % of all cells (histopathology) and CD11b^+^F4/80^+^ cells of leukocytes (FACS) at 12 weeks and 16 weeks. A significantly increased proportion of F4/80^+^ cells in histopathological and FACS analyses indicated augmented inflammation and liver injury in WD mice after 12 weeks of diet. Inflammation was also increased in HFD60 but was not as pronounced as that in WD animals. (c) FACS analysis of CD3^+^ NK1.1^+^ cells at 12 weeks and 16 weeks display a switched (d–f) CD4/CD8 ratio in HFD60 and WD livers after 12 and 16 weeks.

Neutrophils were quantified with Ly6G staining (Figures 4(b), 4(d), and 4(f)). WD livers showed significantly more Ly6G-positive cells than control and HFD60 livers after 12 weeks of diet; whereas at the 16-week timepoint, HFD60 livers presented significantly more neutrophils than control livers (Figure 5(a)). F4/80 staining for macrophages revealed a higher ratio of F4/80-positive cells in WD mice than in the other groups in the 12- and 16-week studies (Figure 5(b)), and these results were confirmed by FACS. The percentage of CD11b^+^/F4-80^+^ cells was significantly increased after 12 weeks of diet in both HFD60 and WD livers and continued to increase after 16 weeks (Figure 5(b)). Differentiation of lymphocytes presented a significantly decreased proportion of CD3^+^ NK1.1^+^ cells in the WD and HFD60 livers compared to those in the control livers at both timepoints (Figure 5(c)). Regarding only the CD3^+^ NK1.1^+^ cells, we observed a transposed CD4/CD8 ratio between the WD and HFD60 livers on the one hand and control livers on the other hand, with a higher ratio of CD8^+^ cells in WD and HFD60 animals after 12 and 16 weeks (Figures 5(d)–5(f)). Histological features of control, HFD60, and WD at both timepoints are summarized in Table 3.

### 3.5. Cafeteria Diet

The final weights of mice fed the CAFD did not differ from those of control animals and were significantly lower than the final weights of HFD60 and WD mice (Supplementary Figure 1a). Compared to control animals, the CAFD mice showed impaired glucose tolerance. Nevertheless, the impairment of glucose tolerance in the HFD60 and WD groups was significantly more pronounced than that in the CAFD group (Supplementary Figure 1a). Furthermore, compared with the control group, the CAFD group showed no differences in metabolism, liver inflammation, or fibrosis. Cholesterol, triglyceride, and ALT serum levels did not differ (Supplementary Figures 1a and b). NAS was comparable between CAFD and control livers, and no signs of fibrosis could be observed (Supplementary Figures 1 b-d).

## 4. Discussion

The literature reports on several available NASH mouse models, each with particular advantages but also certain limitations [5, 15]. As human NASH is heterogeneous in its pathogenesis, no animal model covers all subsets of human NASH.

Therefore, the selection of a NASH model strictly depends on the study hypothesis and is a crucial part of study development. An invalid mouse model leads to frustrating results and the incorrect use of financial and animal resources. In the context of the 3Rs principle, such studies must be strictly avoided. Here, we present a head-to-head comparison of three dietary NASH models including a detailed animal welfare assessment to identify a valid NASH model. Furthermore, several features of NASH are discussed to support other researchers in the process of model selection.

The HFD60 and WD both develop significant overweight, impaired glucose tolerance, and hypercholesterolemia and mimic the metabolic condition of human NASH. Comparing both diets, impaired glucose tolerance is pronounced in HFD60, and even more importantly, HFD60 mice are also insulin resistant. Furthermore, triglyceride levels of the HFD60 mice were significantly higher than those of the WD mice. These results are in line with the literature; increased glucose levels and impaired glucose tolerance as well as hyperlipidemia in high-fat and Western diets are also described by other authors [16, 17].

In addition to an association with metabolic syndrome, histopathological examination of hepatic steatosis and inflammation is crucial for the diagnosis of NASH. Both the HFD60 and WD models show characteristics of NASH, but only in WD livers could NASH be consistently diagnosed in all animals at the 12-week timepoint. Inflammation expressed by higher levels of neutrophils and macrophages was pronounced after feeding WD for 12 weeks but also increased in HFD60 livers in the long-term experiment. In addition to steatosis and hepatic inflammation, the WD had already induced fibrosis after 12 weeks, which progressed after 16 weeks. The literature supports our findings: feeding a high-fat diet 60% regularly induces steatosis but just moderate inflammation or fibrosis [6, 18–20]. Consistent with our WD, Verbeek et al. reported a high-fat high-sucrose diet, which is able to induce NASH with fibrosis after only 12 weeks, together with early obesity and hypercholesterolemia [17]. Savard et al. hypothesized that dietary fat and dietary cholesterol interact synergistically to induce the hepatic features of NASH. Possible mechanisms might be an impaired adiponectin production or the cytotoxicity of free cholesterol [21]. Adiponectin reduces inflammation by inducing the secretion of anti-inflammatory cytokines and inhibiting TNF-*α* and IL-6 [22] whereas free cholesterol can induce perisinusoidal fibrosis through stellate cell activation [23].

However, long-term feeding of HFD60 might also lead to the development of moderate levels of fibrosis [24]. In our study, HFD60 could not influence the fibrosis score, but collagen fibers were increased after feeding HFD60 for 16 weeks compared to control mice.

Importantly, an animal welfare assessment has shown a robust clinical status and no relevant strain in both models. The fur defects were due to physiological rank fights [25] and improved after separating the animals. The fatty, shiny fur observed in a few animals could not be attributed to a lack of coat care and did not affect animals' welfare. The stable clinical status of the mice is crucial for the use of these two models in further studies. In contrast to our experimental diets, some diets, e.g., the methionine-deficient and choline-deficient (MCD) diet, which was still used as a NASH mouse model in 39% of all dietary models published between 2015 and 2017, induce weight-loss and significant welfare impairment [8]. What is particularly alarming is the fact that in almost all studies where MCD is used, no information is given on the clinical condition or mortality of the experimental animals. It is astonishing that a model that impairs animal welfare and lacks the metabolic condition of NASH becomes the standard model of NASH research without any dedicated documentation of the clinical condition. Therefore, we strongly suggest including an animal welfare assessment in every NASH mouse study to comply with international and local institutional standards for ethical experimentation. We hope that consistent documentation and focus on animal welfare will improve future research projects and prevent the use of insufficient mouse models.

In summary, since impaired glucose tolerance, insulin resistance, and hypertriglyceridemia are more pronounced in the HFD60 model but the WD leads to the full spectrum of NASH after only 12 weeks of diet, the HFD60 should be considered for studies focusing on the impact of diabetes and metabolic syndrome on NASH, whereas the WD represents a mouse model of an advanced stage of NASH or even a fibrosis model.

Unfortunately, CAFD could not induce metabolic syndrome or a histopathological diagnosis of NASH. In contrast to our findings, Sampey et al. induced metabolic syndrome and remarkable inflammation in fat and the liver with a similar cafeteria diet in rats [26]. Unlike our CAFD, changing snacks were offered 3 times daily in 2-hour intervals in the rat model, and hyperphagia was triggered. However, in particular consideration of the high workload associated with this model, the CAFD should not be used in NASH studies in mice at least.

A major issue regarding NAFLD and NASH is the progression of hepatic steatosis to inflammation and fibrosis or sometimes even cirrhosis and hepatocellular carcinoma (HCC). The triggers behind this progression are under investigation but still unknown. NKT cells have been shown to play a critical role in regulating innate and adaptive immune responses by not only directly killing target cells but also secreting cytokines [27]. Due to this nature, NKT cells, which can produce different cytokines, are important in controlling liver injury, fibrosis, regeneration, and liver remodeling [28]. However, their role in liver inflammation and fibrosis is complex, and they probably play diverse roles depending on different subsets, the mechanism of activation, and the development of tolerance [29]. Consequently, reports about NKT cells within NASH are controversial. Ji et al. observed a protective role of NKT cells in a fibrosis model [30], in which NKT cell-mediated suppression of hepatic stellate cell (HSC) activation was suggested as a mechanism. These protective effects of NKT cells are in line with our results. We observed a significantly reduced proportion of CD3^+^ NK1.1^+^ cells in the fibrotic livers of WD animals compared to those of control and HFD60 animals. Within the CD3^+^ NK1.1^+^ cell population, the CD4/CD8 ratio decreases in NASH animals with a pronounced proportion of CD8^+^ cells in WD and HFD60 animals. NK1.1^+^ CD8^+^ T cells were reported to be effector cells with innate-like features and prolonged activity in response to microbial pathogens [31] and after allogenic cell transplantation [32]. Hence, it can be assumed that the CD8^+^ NK1.1^+^ cell population in our study encompasses effector cells with a cytotoxic and profibrotic phenotype.

In contrast to our findings, NKT cells have a pathogenic role in liver inflammation in many experimental models of NAFLD [33, 34]. Wolf et al. observed a significant increase in hepatic CD3^+^ NK1.1^+^ cells in choline-deficient high-fat diet-fed mice. They concluded that NKT cells enhance steatosis, NASH development, and the transition to HCC [35]. However, different experimental diets/study settings make it difficult to compare the results. Further studies should respect the variances in different NASH and fibrosis models as well as a consistent definition of NKT cells.

## 5. Conclusions

In conclusion, the WD and HFD60 can be recommended as dietary NASH mouse models in C57/B6 mice. Whereas the WD is suitable for NASH studies focusing on inflammation and fibrosis, the HFD60 is a NAFLD model recommended for studies concentrating on metabolism. Based on our data, the CAFD is not recommended in C57/bl6 mice. Furthermore, NKT cells are important players in NASH pathology. We observed a reduction of CD3^+^ NK1.1^+^ cells in NASH livers. Within the population of these CD3^+^ NK1.1^+^ cells, effector cells dominate in WD and HFD60 livers leading to the assumption that protective NKT cells are decreased and the remaining population has mostly cytotoxic and profibrotic effects.

## Figures and Tables

**Table 1:** Design of score sheet.

Parameter		Score
*General state*		
Body weight	Variation < 5%	1
	Weight reduction 5-10%	5
	Weight reduction 11-20%	10
	Weight reduction > 20%	20
Fur	Fur faulty (reduced body hygiene)	1
	Fur lusterless and disheveled	5
	Fur dirty	10
Stoma	Scruffy	1
	Sticky or moist	5
Eyes	Turbid	5

*Behavior*		
Nutrition	Hypophagia	5
	Fasting	10
Activity	Limited motor function or hyperkinetic	5
	Ducked posture, lethargy, coordination disorder	10
Social behavior	Self-isolation	5
	Missing fight-or-flight response	10
	Self-amputation (automutilation)	20

*Clinical results*		
Digestion	Diarrhea	5
	Steatorrhea	5
Temperature, pulse, and respiration	Variation of temperature 1–2°C, respiration, and pulse ±30%	5
	Variation of temperature > 2°C, respiration, and pulse ±50%	20
Vegetative symptoms	Shivering	10
Trial-specific indicators	Edema	20
	Ascites	20
	Jaundice	20

**Table 2:** Degree of strain.

DS	Category	Procedure	Score
DS0	No strain		0
DS1	Minor category	Careful observation necessary	1-4
DS2	Moderate category	Supportive measures (additional analgesia or fluids)	5-9
DS3	Critical category	DS2 + veterinary support	10-19
DS4	High-grade strain	Consult animal protection commissary, veterinary support necessary, termination of experiment, euthanasia	≥20

**Table 3:** Histological features of control, HFD60, and WD.

		12 weeks			16 weeks		
		Control	HFD60	WD	Control	HFD60	WD
*NAS*	NAS	1 ± 0.89	3.67 ± 1.03	5.83 ± 0.75	0.83 ± 0.41	4.33 ± 1.21	5.5.±1.64
	Steatosis	0	1.33 ± 0.52	3	0	1.33 ± 0.52	2.67 ± 0.52
	Inflammation	1.0 ± 0.89	0.83 ± 0.41	1.83 ± 0.75	0.83 ± 0.41	2	1.83 ± 0.75
	Ballooning	0	1.5 ± 0.55	1	0	0.83 ± 0.75	1.0 ± 0.63

*Fibrosis*	Fibrosis score	0	0.17 ± 0.41	1.5 ± 0.55	0.5 ± 0.55	1.2 ± 0.41	2.7 ± 1.5
	Sirius red	0.62 ± 0.13	1.01 ± 0.579	1.79 ± 0.99	0.73 ± 0.2	1.27 ± 0.41	2.24 ± 0.41

*Inflammation*	F4/80	17 ± 5.2	30 ± 6.9	38 ± 2.4	12 ± 2.2	26 ± 10	31 ± 5.3
	Ly6G	6.7 ± 1.3	9.6 ± 2.69	15.4 ± 4.53	6.56 ± 1.09	10.63 ± 1.9	8.33 ± 1.24

## Data Availability

The data used to support the findings of this study are available from the corresponding author upon request.

## Supplementary Materials

Supplementary Materials Supplementary Table 1: timetable CAFD. Supplementary Figure 1: cafeteria diet. Supplementary Figure 2: dot-plot diagram of ALT levels.
