# Supplementary material for: Mouse Models of Nonalcoholic Steatohepatitis: Head-to-Head Comparison of Dietary Models and Impact on Inflammation and Animal Welfare
Source: Gastroenterol Res Pract. 2020 Jul 13;2020:7347068. doi: 10.1155/2020/7347068 (PMC7374209; doi:10.1155/2020/7347068)
Supplement: Supplementary Materials — Supplementary Table 1: timetable CAFD. Supplementary Figure 1: cafeteria diet. Supplementary Figure 2: dot-plot diagram of ALT levels. [file 7347068.f1.pdf]

Supplementary Materials

Supplementary Table 1: Timetable CAFD

Day	Values are per 1 g of food item	Total kcal/g	Total fat g	Total carbo-hydrate g	Sugars g	Protein g
1	BiFi®(LSI-Germany GmbH, Germany)	5.1	0.45	0.01	0.009	0.25
2	Chio Flips Classic Peanut (Intersnack Switzerland Ltd., Switzerland)	4.89	0.24	0.49	0.03	0.14
3	Ültje salted peanuts (Intersnack Switzerland Ltd., Switzerland)	6.22	0.57	0.13	0.05	0.25
4	K-Classic Soft Cookies Triple Chocolate (Kaufland GmbH & Co. KG, Germany)	5.02	0.25	0.61	0.36	0.067
5	Snickers (Mars Inc., McLean, USA)	4.74	0.23	0.63	0.51	0.076

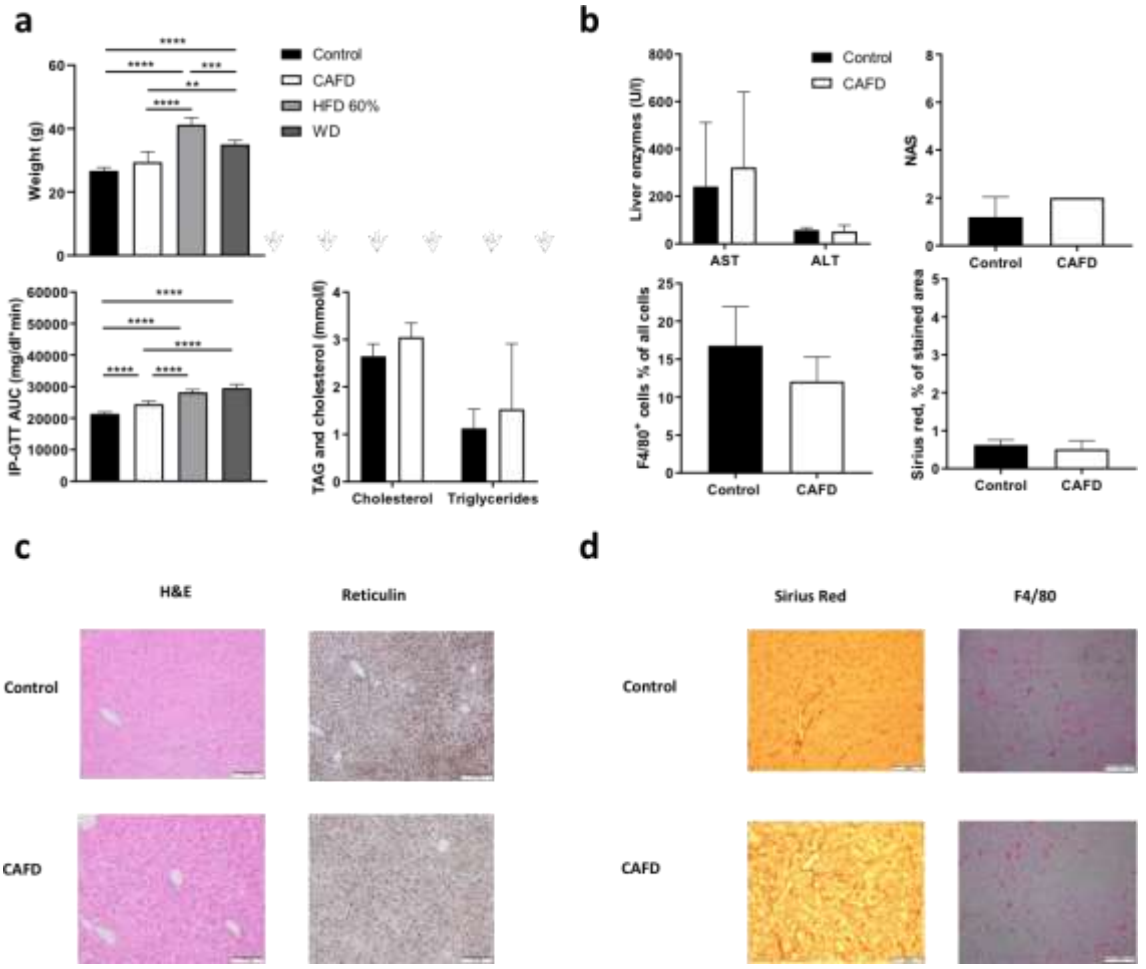

Supplementary Figure 1: Cafeteria Diet

(a) Body weight, IP-GTT, TAG and Cholesterol after 12 weeks of diet. Total body weight was significantly lower in CAFD mice than in HFD60 and WD mice. The area under the glucose time curve displays impaired glucose tolerance in CAFD mice compared to that of the control mice but on a significantly lower level than that of HFD60 and WD mice. TAG and cholesterol levels showed no differences between CAFD and control animals. (b) Serum ALT, NAS, F4/80^{+} cells % of all cells and Sirius Red (histopathology) were not different between CAFD and control mice. (c) Representative images of H&E- and reticulin-stained liver sections. (d) Representative images of Sirius red and F4/80 stained liver sections. Magnification 40x, scale bars 200 µm.

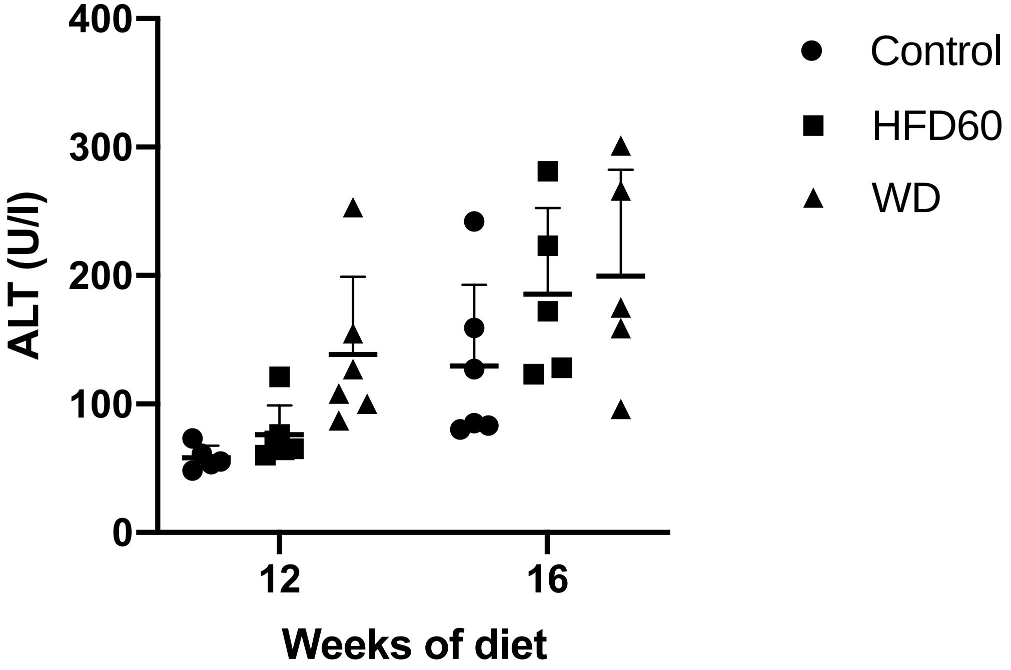

400
300
200
100
0
ALT (U/l)
12
16
Weeks of diet
Control
HFD60
WD
